# Taldefgrobep Alfa and the Phase 3 RESILIENT Trial in Spinal Muscular Atrophy

**DOI:** 10.3390/ijms251910273

**Published:** 2024-09-24

**Authors:** Laurent Servais, Lindsey Lee Lair, Anne M. Connolly, Barry J. Byrne, Karen S. Chen, Vlad Coric, Irfan Qureshi, Susan Durham, Daniel J. Campbell, Grant Maclaine, Jackie Marin, Clifford Bechtold

**Affiliations:** 1 Department of Pediatrics, University of Oxford, Oxford OX3 9DU, UK; 2 Division of Child Neurology, Department of Paediatrics, Centre de Référence des Maladies Neuromusculaires, University Hospital of Liège, University of Liège, Boulevard Du 12e De Ligne, 4000 Liege, Belgium; 3 Biohaven Pharmaceuticals Inc., New Haven, CT 06510, USA; 4 Nationwide Children’s Hospital, Columbus, OH 43205, USA; 5 Department of Pediatrics, University of Florida, Gainesville, FL 32611, USA; 6 Spinal Muscular Atrophy Foundation, 970 W Broadway STE E, PMB 140, Jackson, WY 83001, USA

**Keywords:** taldefgrobep, myostatin, spinal muscular atrophy, myostatin inhibitor, SMN upregulation, phase 3 clinical trial, RESILIENT, antimyostatin, SMA, SMN upregulator

## Abstract

Spinal muscular atrophy (SMA) is a rare, genetic neurodegenerative disorder caused by insufficient production of survival motor neuron (SMN) protein. Diminished SMN protein levels lead to motor neuron loss, causing muscle atrophy and weakness that impairs daily functioning and reduces quality of life. SMN upregulators offer clinical improvements and increased survival in SMA patients, although significant unmet needs remain. Myostatin, a TGF-β superfamily signaling molecule that binds to the activin II receptor, negatively regulates muscle growth; myostatin inhibition is a promising therapeutic strategy for enhancing muscle. Combining myostatin inhibition with SMN upregulation, a comprehensive therapeutic strategy targeting the whole motor unit, offers promise in SMA. Taldefgrobep alfa is a novel, fully human recombinant protein that selectively binds to myostatin and competitively inhibits other ligands that signal through the activin II receptor. Given a robust scientific and clinical rationale and the favorable safety profile of taldefgrobep in patients with neuromuscular disease, the RESILIENT phase 3, randomized, placebo-controlled trial is investigating taldefgrobep as an adjunct to SMN upregulators in SMA (NCT05337553). This manuscript reviews the role of myostatin in muscle, explores the preclinical and clinical development of taldefgrobep and introduces the phase 3 RESILIENT trial of taldefgrobep in SMA.

## 1. Introduction

Spinal muscular atrophy (SMA) is a rare autosomal recessive disorder with a reported annual incidence of 1 in 6000 to 1 in 30,000 live births [1]. A homozygous deletion at chromosome 5q13, which codes for the survival motor neuron 1 (*SMN1*) gene, causes 95% of SMA cases [2]. *SMN1* point mutations, deletion in the other *SMN1* allele, or, very rarely, biallelic small-scale mutations in *SMN* exons can be identified in the other cases of SMA [3]. Individuals with SMA express diminished levels of survival motor neuron (SMN) protein, leading to motor neuron loss and associated muscle weakness due to muscle atrophy [2]. Affected individuals rely on the *SMN2* gene to create functional SMN protein. Because *SMN2* generates only 10% of the overall SMN level, there is a correlation between the number of copies of *SMN2* in a given patient and clinical severity; children with a larger number of *SMN2* copies have milder disease [4,5].

Molecular genetic testing confirms a diagnosis of SMA [6]. Individuals are usually diagnosed genetically, either via newborn screening or on the basis of a family history of SMA in the presence of symptoms [3]. Classically, SMA has been categorized into 5 types (type 0 to type 4) based on differences in phenotypic expression related to age at symptom onset and the maximal motor milestone achieved without treatment [3,7]. Individuals with SMA type 0 or type 1 have the earliest onset, characterized by factors such as fetal demise or early postnatal clinical findings. Persons with SMA type 4 have a much later onset of disease, with symptoms manifesting during adulthood [3,7,8].

The recent regulatory approval and use of three disease-modifying therapies that increase SMN protein production (SMN upregulators) have significantly altered the course of disease in SMA. Nusinersen is a *SMN2*-directed antisense oligonucleotide administered intrathecally [9,10]. Onasemnogene abeparvovec is an adeno-associated virus, vector-based gene therapy indicated in patients less than 2 years of age who have SMA and bi-allelic mutations in the *SMN1* gene [11,12]. Risdiplam, an *SMN* splicing modifier, is orally administered once daily [13,14]. These agents were approved by the US Food and Drug Administration (FDA) in 2016, 2019 and 2020, respectively, with subsequent approvals by the European Medicines Agency and other global regulatory bodies.

Despite currently approved therapies for SMA, a high unmet need persists [15,16,17,18]. Pivotal trials that supported the approval of currently available therapies for SMA demonstrate that although current standard-of-care treatments are effective in helping patients achieve milestones that they would not have otherwise achieved and improve survival, functional deficits remain in treated patients across the spectrum of motor milestones. Functional, respiratory, caregiver and other supports are still needed [9,13,15,17,19,20,21]. 

The clinical improvements seen with these new treatments have led clinicians to rely on functional status, age at treatment initiation, number of *SMN2* copies and age at symptom onset, rather than the classical SMA subtypes, to define clinical SMA phenotypes [22,23]. Infants are now being diagnosed and have access to treatment soon after birth. SMN upregulators have demonstrated valuable efficacy by helping patients achieve developmental milestones and improving survival with SMA. 

With the availability of SMN upregulators, it is theoretically expected that patients will not only maintain their current functional status but also experience better motor function and quality of life in the long term. However, while most infants with SMA are being diagnosed and treated early, many children and adults lose considerable motor function and muscle strength before their diagnosis and initiation of SMA treatment. Furthermore, no matter when treatment is initiated, many individuals with SMA will continue to experience reduced levels of functioning, significant weakness and decreased quality of life while on SMN upregulator therapy [13,19,24,25,26,27]. Patients and families have reported that gaining muscle strength and achieving new motor function are among their top priorities for future research and therapies [25]. Even small gains that enhance independence can improve quality of life. 

The combination of myostatin inhibition and SMN upregulation holds promise as a therapeutic strategy in SMA by offering a two-pronged approach that targets the whole motor unit via mechanisms of action that include: (1) optimizing SMN protein and directly restoring motor neuron function with SMN upregulators and (2) reversing muscle atrophy by inhibiting the myostatin and activin A pathway. 

This manuscript explores the role of myostatin in muscle, highlights the preclinical and clinical development of the myostatin inhibitor, taldefgrobep alfa and introduces the phase 3 RESILIENT trial of taldefgrobep alfa in SMA. The RESILIENT trial is uniquely designed to evaluate the novel mechanism of taldefgrobep alfa that enables patient friendly administration and includes patients based on function with all forms of SMN upregulation, reflecting the current standards of SMA therapy.

## 2. Myostatin and Muscle Growth

**Figure 1:**
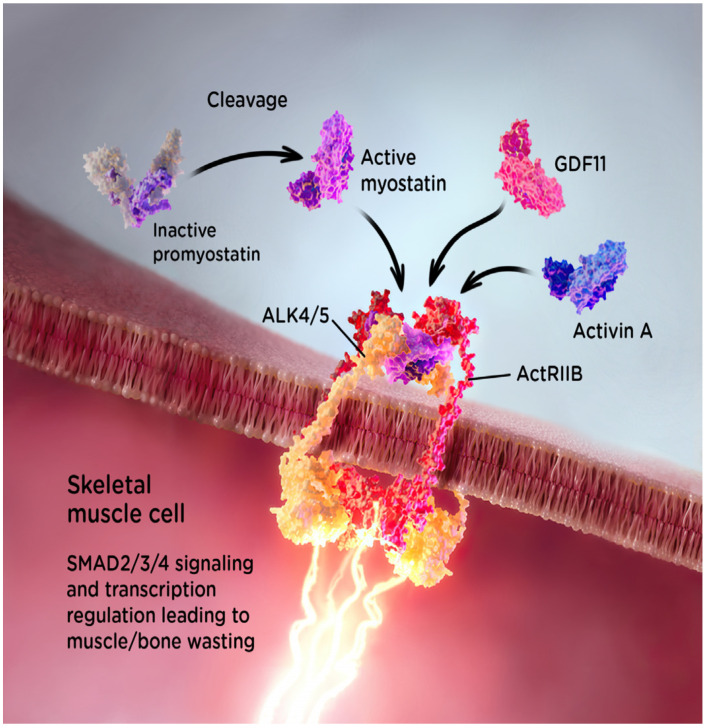
Myostatin mechanism of action in skeletal muscle. ActRIIB, activin receptor type IIB; ALK4, activin type I receptor-like kinase type 4; ALK5, activin type I receptor-like kinase type 5; GDF11, growth differentiation factor 11.

Myostatin, a paracrine signaling molecule of the transforming growth factor beta (TGF-β) superfamily, is initially produced in the form of an inactive precursor called promyostatin, which is subsequently processed into the active mature form [28,29,30,31,32]. Encoded by the myostatin (*MSTN*) gene and expressed primarily by skeletal muscle cells, mature myostatin acts as a negative regulator of muscle growth via activin receptor complexes and a mechanism that has been suggested to involve a reduction in myogenesis [29]. The activin receptor complex consists of membrane-bound type I and type II receptors that are made up of an extracellular ligand-binding domain, a single transmembrane domain and an intracellular serine kinase domain [33]. Myostatin binds to activin receptor type IIB (ActRIIB), forming a complex that activates activin type I receptor-like kinase types 4 (ALK4) and type 5 (ALK5), thereby signaling the Smad2/3/4 pathway to inhibit myogenesis and resulting in muscle wasting and atrophy [29,33,34,35]. Figure 1 presents an illustration of myostatin binding and signaling in skeletal muscle. 

Since the discovery of myostatin, researchers have explored ways to suppress its activity with the goal of combatting muscle atrophy [29,36]. For more than two decades, molecules that block the myostatin signaling pathway have been investigated in a broad range of muscle diseases, including facioscapulohumeral muscular dystrophy, Becker muscular dystrophy, limb-girdle muscular dystrophy, Duchenne muscular dystrophy (DMD) and inclusion body myositis. Unfortunately, significant improvements in muscle function or strength have not been realized [37,38,39,40,41,42]. Underlying muscle pathology, concomitant high-dose steroid use and low levels of circulating myostatin may have contributed to the lack of functional improvements seen with myostatin inhibitors in these diseases [37].

However, SMA is a disorder with the potential to benefit from a therapeutic approach utilizing the myostatin pathway. The causative pathology in SMA is insufficient SMN protein, fostering neuronal insufficiency and serving as a primary driver of skeletal muscle atrophy [43,44,45]. Not all muscles are affected and the muscle structure remains intact, with muscle atrophy directly correlated with the degree of muscle innervation. Muscles innervated by nerves that are less influenced by loss of SMN exhibit less atrophy [46]. With the approval of disease-modifying therapies to address this deficiency, an opportunity exists to reverse skeletal muscle atrophy in SMA by using muscle-targeted agents, such as myostatin/activin A inhibitors, in combination with SMN upregulation [47].

This hypothesis is supported by animal models of SMA that demonstrate neurological complications and limited life span without intervention. Similar to human SMA disease, the SMA mouse models (SMN∆7 or Taiwanese SMA model) have shown significant improvement in survival and function when treated with SMN upregulators [46,48]. In these studies, coadministration of a myostatin inhibitor along with SMN upregulation extended the benefits, with increases in muscle mass, the size of sensory neurons in the dorsal root ganglia and overall life span [46,48]. Unlike other muscular degenerative diseases, the atrophy of intact muscle in SMA presents a unique opportunity for interventions that induce hypertrophy. Collectively, the mechanisms of action and data from these studies support the rationale for considering myostatin inhibition in combination with SMN upregulating therapy as a therapeutic intervention in SMA.

## 3. Taldefgrobep Alfa

**Figure 2:**
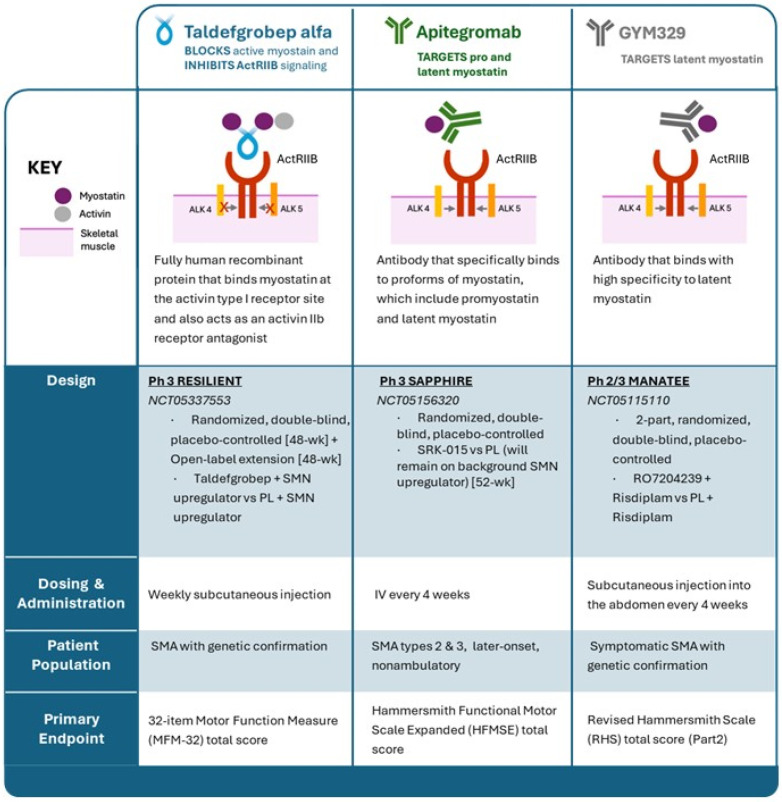
Myostatin inhibitors in late-stage clinical trials in SMA. HFMSE, Hammersmith Functional Motor Scale Expanded; IV, intravenous; MFM-32, 32-item Motor Function Measure; Ph, phase; PL, placebo; RHS, Revised Hammersmith Scale; SMA, spinal muscular atrophy; SMN, survival motor neuron.

Taldefgrobep is part of a unique class of molecules that inhibit activation of the activin A receptor and directly impact downstream signaling. Many inhibitors evaluated target myostatin directly, but there are limited attempts to block signaling at the receptor level [49]. Although there are three myostatin inhibitors in clinical development in Phase 2/3 clinical trials involving individuals with SMA, only taldefgrobep specifically targets signaling through the receptor with competitive inhibition of activin A and myostatin (Figure 2).

**Figure 3:**
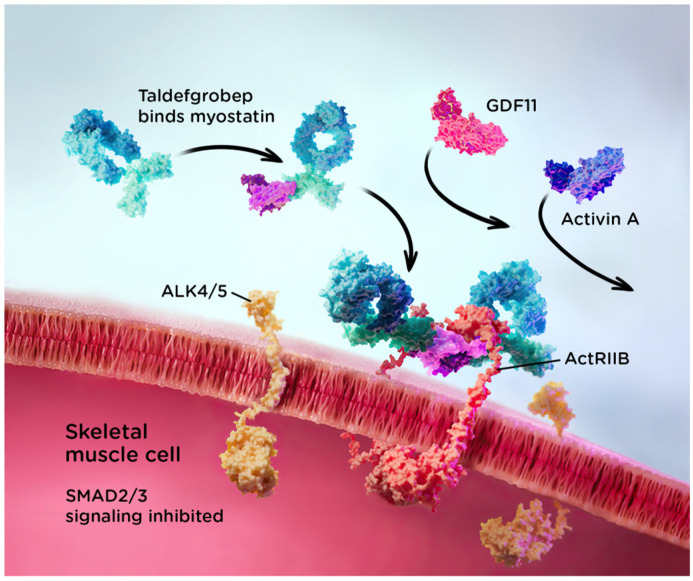
Taldefgrobep alfa mechanism of action [53,54].

Taldefgrobep is a novel, fully human recombinant protein specifically designed to selectively bind to myostatin and act as a competitive inhibitor of ligands that signal through the activin II receptor. As a fusion protein, taldefgrobep was designed to have optimal affinity for myostatin but avoid off-target activity with molecules in the TGF-β pathway that have negative safety signals. By blocking the formation of the myostatin-activin receptor complex, taldefgrobep prevents downstream activity that leads to muscle atrophy. This receptor blockade also inhibits activin A binding and signaling in tissue in which myostatin is active [50,51]. Taldefgrobep offers a unique mechanistic approach that potentially minimizes off-target effects resulting from activin type II receptor blockage in nonmuscular tissue and reduces the capacity for wasting in muscular tissue, which could result from activin A signaling if myostatin alone were inhibited [52]. Figure 3 depicts the mechanism of action of taldefgrobep. 

Modified with a human IgG1 Fc tail to prolong half-life in circulation, taldefgrobep has high in vivo potency, high affinity and favorable pharmacokinetics that allow for subcutaneous (SC) administration. Additionally, taldefgrobep has the capacity to be administered in various body sites, including the arm, abdomen, or thigh, with comparable bioavailability. 

### 3.1. Preclinical Studies of Taldefgrobep in SMA

Taldefgrobep and the SMN protein upregulator SMN-C1 were evaluated as a combined therapeutic approach in two different preclinical studies of murine SMA models using SMNΔ7 mice. Both studies included SMNΔ7 and wild-type mice as controls with the primary aim of evaluating differences in muscle morphometrics and function between taldefgrobep-treated SMA mice and control SMA mice. 

In the first preclinical study (RK050216), taldefgrobep (10 mg/kg) was administered to an experimental group of nine mice from postnatal day 24 (PND24) through PND52. Additionally, low-dose SMN-C1 (0.1 mg/kg) was provided beginning on PND1, followed by high-dose SMN-C1 (3 mg/kg) from PND24 to PND52. A total of 10 SMA control mice also received SMN-C1 per the same dosing schedule. In a second preclinical study (RK100115), an experimental group of 20 mice were given taldefgrobep (10 mg/kg) from PND21 to PND42 along with low-dose SMN-C1 (0.1 mg/kg) from PND2 to PND62; 15 SMA control mice received SMN-C1 with the same dosing schedule. 

**Figure 4:**
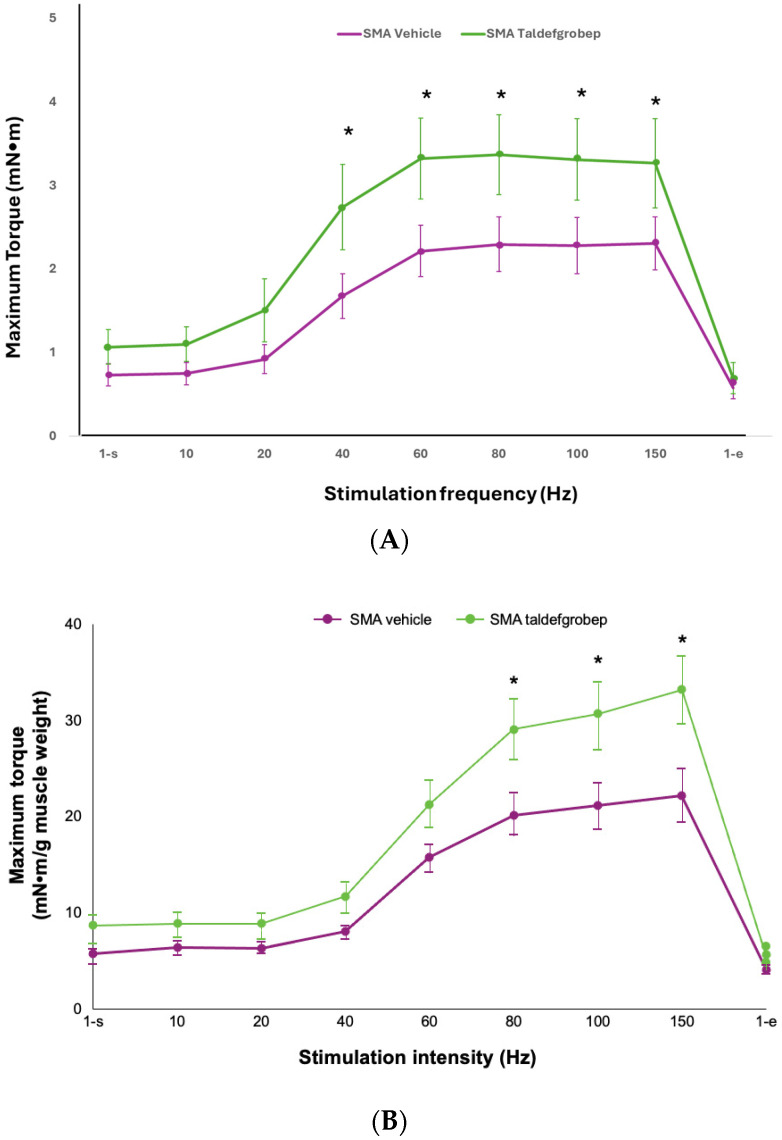

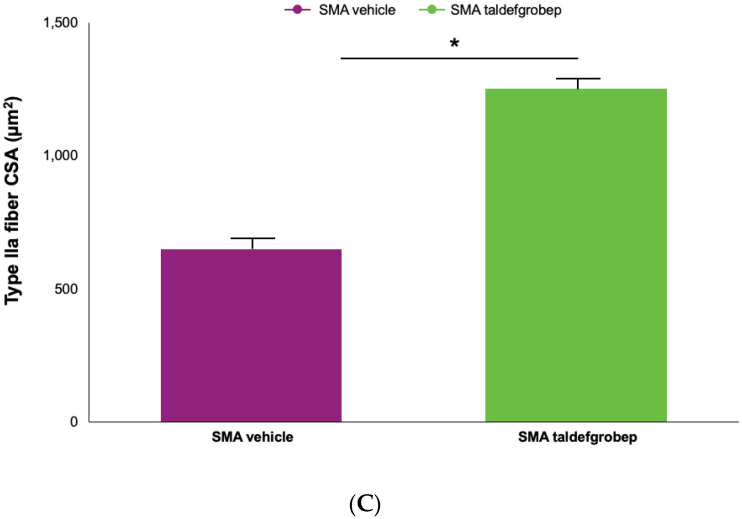
(**A**) Plantar flexor muscle function at PND52, based on maximum torque in the RK050216 study. * *p* < 0.05 (using post hoc Holm–Sidak tests for pairwise comparisons) for SMA mice treated with the combination of SMN-C1 (3 mg/kg) and taldefgrobep (10 mg/kg) vs. SMA mice treated with SMN-C1. PND52, postnatal day 52. (**B**) Gastrocnemius muscle function in the RK100115 preclinical study. Muscle performance at PND48, based on maximal torque normalized to gastrocnemius weight in SMA mice treated with the combination of low-dose SMN-C1 (0.1 mg/kg) and taldefgrobep (10 mg/kg) vs. SMA mice treated with low-dose SMN-C1 and vehicle. * *p* = 0.01 at 80 Hz; *p* = 0.01 at 100 Hz; *p* = 0.02 at 150 Hz (using post hoc Holm–Sidak tests for pairwise comparisons following a 2-way repeated measures ANOVA). ANOVA, analysis of variance; PND48, postnatal day 48. (**C**) Ref. [5] Type IIa muscle fiber CSAs in the RK100115 preclinical study at PND48. * *p* < 0.05 (using a 1-way ANOVA) for SMA mice treated with the combination of low-dose SMN-C1 (0.1 mg/kg) and taldefgrobep (10 mg/kg) vs. SMA mice treated with low-dose SMN-C1 alone. CSA, cross-sectional area [5,55,56,57].

In the RK050216 study, plantar flexor muscle fiber type composition and overall cross-sectional area (CSA) as well as masseter muscle function were similar between taldefgrobep-treated SMA mice and control SMA mice at PND52. However, at PND52, the combination of taldefgrobep and high-dose SMN-C1, as compared to SMN-C1 alone, resulted in improved plantar flexor muscle function based on maximum torque improvements at stimulation frequencies of 40 Hz, 60 Hz, 80 Hz and 100 Hz (*p* < 0.05 each, using pairwise comparisons made by the Holm-Sidak method) (Figure 4A). Additionally, by PND52, gains in gastrocnemius muscle weight (*p* = 0.08, using 1-way analysis of variance (ANOVA) and the mean plantar flexor muscle fiber CSA (*p* = 0.14, using 1-way ANOVA) were higher in the combination group than with SMN-C1 alone, although these differences were not statistically significant. 

In the RK100115 study, the combination of taldefgrobep and low-dose SMN-C1 was associated with several improvements in muscle weight and/or function compared to SMN-C1 alone, including significantly increased body weight at PND48 (*p* < 0.05, using the Holm–Sidak method) and increased gastrocnemius muscle weight at PND62 (*p* < 0.05, using the Holm–Sidak method). Improvements were also seen at PND48 and/or PND62 across several metrics of gastrocnemius muscle performance and contraction and/or relaxation kinetics. At PND48, maximal torque normalized to gastrocnemius weight was higher in SMA mice treated with the combination of low-dose SMN-C1 and taldefgrobep vs SMA mice treated with low-dose SMN-C1 and vehicle (*p* = 0.01 at 80 Hz; *p* = 0.01 at 100 Hz; *p* = 0.02 at 150 Hz, using post hoc Holm–Sidak tests for pairwise comparisons following a 2-way repeated measures ANOVA) (Figure 4B). 

At PND62, the taldefgrobep and SMN-C1 combination was also associated with improved maximal force in the masseter muscle at 150 Hz (*p* = 0.03, using post hoc Holm–Sidak tests for pairwise comparisons following a 2-way repeated measures ANOVA). However, both maximal force normalized to body weight (*p* = 0.11, using post hoc Holm–Sidak tests for pairwise comparisons following a 2-way repeated measures ANOVA) and maximum rate of relaxation at 150 Hz (*p* = 0.05, using 1-way ANOVA) improved numerically with the combination treatment, although these differences were not statistically significant. Differences in muscle fiber on cross-section were also observed in this study. SMA mice treated with the combination exhibited increased mean muscle fiber CSA at PND48 (*p* < 0.05, using 1-way ANOVA), increased type IIb muscle fiber CSA at PND48 (*p* < 0.05, using 1-way ANOVA) and restoration of type IIa atrophic muscle fibers at both PND48 and PND62 (*p* < 0.05, using 1-way ANOVA), compared to mice that received SMN-C1 alone. Figure 4C presents type IIa muscle fiber CSAs at PND48. 

In summary, both SMA mouse model studies demonstrated a benefit of combining taldefgrobep with an SMN upregulator for improved muscle function. The RK050216 study in mice demonstrated improvements in gastrocnemius muscle size and plantar flexor muscle fiber CSA with combination therapy compared to SMN upregulation alone. Additionally, this study concluded that a treatment effect may be influenced by the duration or timing of dosing. Results from the RK100115 study revealed multiple significant differences between treatment groups, ranging from increased body weight to improved muscle function and increased type IIa and IIb myofiber size with the combination treatment compared to the low-dose SMN upregulator alone. By PND62, the smaller population size due to mortality may have affected the results at this timepoint; however, data regarding the masseter suggest that a longer duration of taldefgrobep treatment may be required for a discernable effect in this muscle, which is particularly vulnerable in SMA. Thus, these two preclinical studies support the advancement of taldefgrobep in combination with SMN protein upregulation agents as potential treatment for SMA.

### 3.2. Taldefgrobep in the Clinical Setting

#### Healthy Adults

Taldefgrobep has been tested in two randomized phase 1 trials in healthy adults to determine its safety, tolerability, pharmacodynamics, pharmacokinetics, bioavailability and immunogenicity. In total, these studies enrolled 216 healthy adults, of whom 179 received taldefgrobep and 37 received placebo [41,58,59]. Participants were 18 to 55 years of age; the women were not of childbearing potential. The clinical profiles of the participants did not significantly deviate from the normal range with regard to physical examinations, medical history, laboratory findings and electrocardiograms (ECGs) [41,58,59]. Participants received up to 180 mg of SC taldefgrobep once weekly (Q1W) or 45 mg SC taldefgrobep every two weeks [41].

Taldefgrobep was found to be safe and well tolerated in both the initial study (NCT02145234), which tested single and multiple dose strategies to determine optimal taldefgrobep dosing and the second study (NCT03100630), which assessed how SC administration of taldefgrobep in the arm, abdomen, or thigh affected clinical bioavailability [58,59]. In both studies, there were no deaths, adverse events (AEs), or serious adverse events (SAEs) that led to discontinuing treatment, nor were there clinically significant changes in ECG parameters, laboratory findings, or vital signs. Antidrug antibodies did not appear to affect the safety or exposure of taldefgrobep in this population.

**Figure 5:**
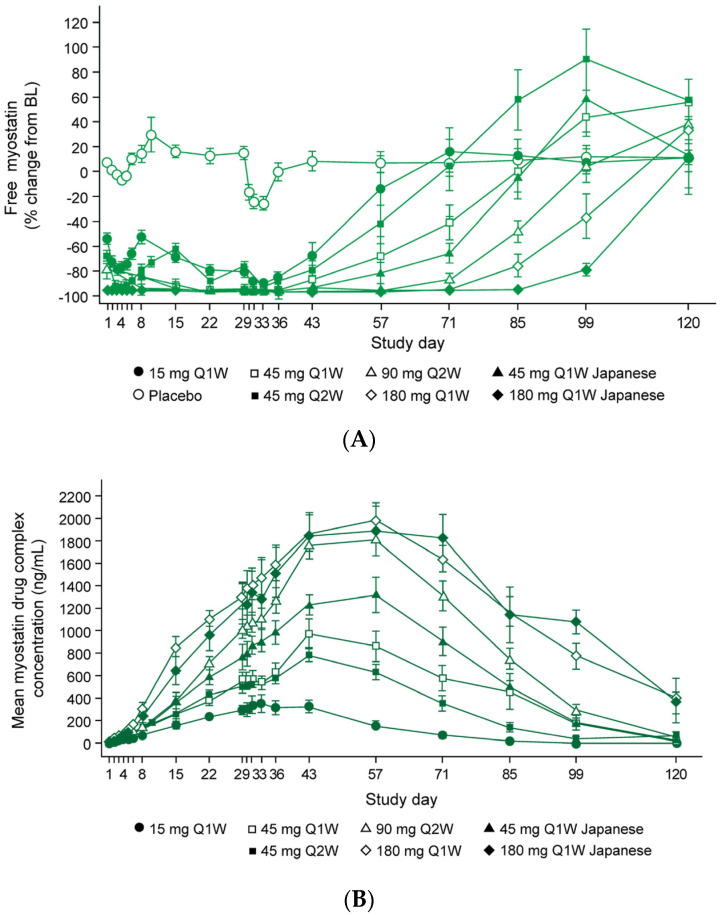
Taldefgrobep alfa in healthy adults. (**A**) Free myostatin levels. (**B**) Ref. [41] Taldefgrobep-myostatin complex concentrations. BL, baseline; Q1W, once weekly; Q2W, twice weekly [41].

In the multiple ascending dose (MAD) arm of the initial study, mean serum taldefgrobep concentrations over time demonstrated dose-dependent increases in taldefgrobep exposure. At day 22, the maximum reduction of free myostatin was at least 90% for all doses (Figure 5A). Over the course of the study, maximum concentrations of total myostatin ranged from 300 ng/mL to 2000 ng/mL. Marked lowering of serum taldefgrobep concentration occurred after administration of taldefgrobep 45 mg Q1W and the drug-myostatin complex was detectable for weeks after dosing stopped (Figure 4A,B) [41,60].

**Figure 6:**
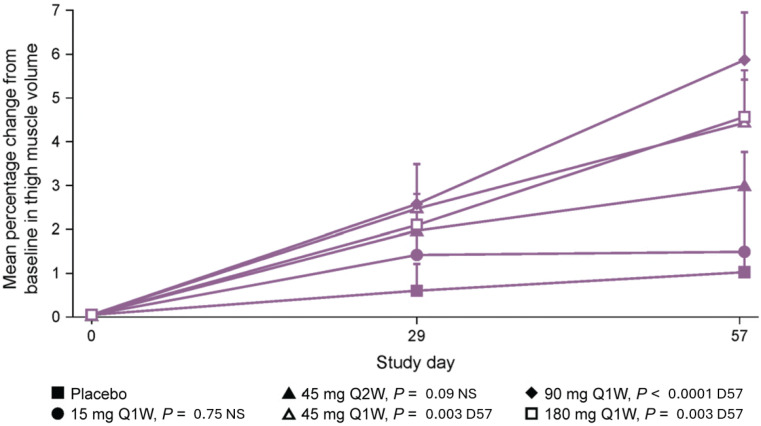
MAD phase of taldefgrobep study in healthy volunteers. Change in thigh muscle volume over time [41]. D57, day 57; MAD, multiple ascending dose; NS, not statistically significant [41].

Additionally, during the MAD component of the initial phase 1 study, participants receiving taldefgrobep at all doses experienced numerical increases in right thigh muscle volume at day 29 and day 57 compared to participants receiving placebo (Figure 6). Statistically significant increases from baseline were observed at day 57 in participants who received the higher taldefgrobep doses (45 mg, 90 mg and 180 mg Q1W), with mean increases ranging from 3.41% with 45 mg (*p* = 0.003; n = 11) and 3.52% with 180 mg (*p* = 0.003; n = 10) to 4.75% with 90 mg (*p* < 0.0001; n = 9). Analysis of these data was based on a mixed model for repeated measures, with treatment, visit and treatment-by-visit interaction as fixed effects and measurements within participants as repeated measures [41].

During the MAD phase, 60% of all participants receiving taldefgrobep (n = 72) and 44% of participants receiving placebo (n = 25) experienced AEs, with injection site erythema (12%, n = 12) and upper respiratory tract infection (11%, n = 11) being the most frequently occurring AEs. Except for two unrelated moderate AEs involving vomiting and bacterial sinusitis, all AEs were mild. No SAEs were reported.

The bioavailability of taldefgrobep and the change in free myostatin concentration from baseline were comparable for each of the three sites of administration (arm, abdomen and thigh). After SC administration of a single 50 mg dose of taldefgrobep in the abdomen, arm, or thigh, bioavailability was similar across injection sites; the assessment of bioavailability was based on maximum plasma concentration, area under the concentration-time curve from time zero to the last measurable concentration and area under the plasma concentration-time curve extrapolated to infinity. The median time to maximum plasma concentration was greater for administration in the arm (120 h) or thigh (120 h) vs the abdomen (72 h). The change in free myostatin concentration was similar for injections administered in the abdomen, arm, or thigh.

These encouraging results led to the clinical investigation of taldefgrobep in more than 200 participants with DMD, further establishing the tolerability of taldefgrobep [41]. This investigation included a phase 1b/2 multisite, randomized, placebo-controlled, MAD study of SC taldefgrobep in 43 boys aged 5 to <11 years who were diagnosed with DMD and were ambulatory without assistance. Magnetic resonance imaging data revealed that at the end of the 24-week placebo-controlled phase, a 5.45% increase in contractile CSA from baseline occurred in participants treated with taldefgrobep compared to a 0.79% reduction in contractile CSA among those receiving placebo [41]. At 168 weeks, there was a 3.7% increase and 2.2% decrease in contractile CSA from baseline in the taldefgrobep- and placebo-treated populations, respectively [41]. Correspondingly, at 168 weeks, participants who received taldefgrobep vs. placebo experienced increases in noncontractile CSA of 9.7 cm^2^ and 10.5 cm^2^, respectively [41].

## 4. RESILIENT

**Figure 7:**
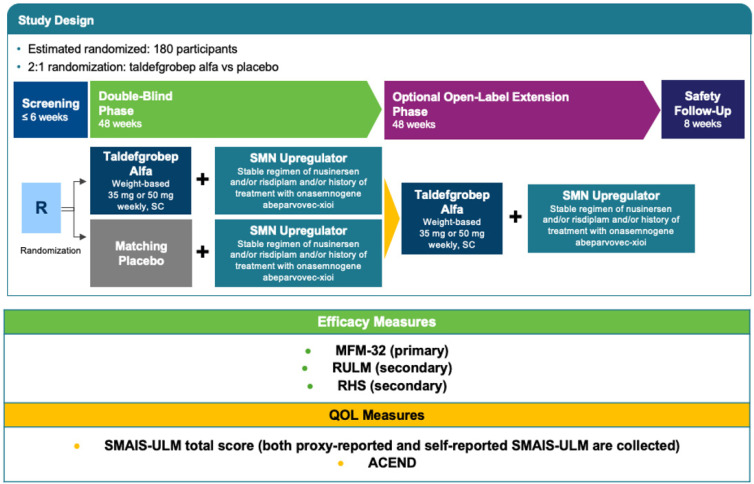
Phase 3 RESILIENT study design. ACEND, Assessment of Caregiver Experience with Neuromuscular Disease; QOL, quality of life; RULM, Revised Upper Limb Module; SC, subcutaneous; SMAIS-ULM, SMA Independence Scale–Upper Limb Module [5,53,54,55,56,57].

Driven by the high unmet need for enhanced treatment in SMA and given the body of data on taldefgrobep—including its demonstrated inhibition of myostatin and activin A in muscle, preclinical data and an extensive safety profile in healthy adults and pediatric participants with neuromuscular disease—investigators initiated the RESILIENT clinical trial (NCT05337553) in 2022 [61]. RESILIENT is a multicenter, phase 3, randomized, double-blind, placebo-controlled study designed to assess safety and efficacy of taldefgrobep alfa in participants with 5q autosomal recessive SMA who are on a stable regimen of nusinersen and/or risdiplam and/or who have a history of receiving onasemnogene abeparvovec, regardless of their SMA type or ambulatory status [5,62]. Figure 7 provides a schematic overview of the RESILIENT study design.

### 4.1. Methods

#### 4.1.1. Study Setting and Study Population

Enrollment for RESILIENT occurred at 53 sites (including hospitals as well as research and academic centers) in 9 countries: Belgium, the Czech Republic, Germany, Italy, the Netherlands, Poland, Spain, the UK and the US (full list of sites is available at ClinicalTrials.gov, NCT05337553) [5,51,61].

In developing RESILIENT, investigators were guided by their commitment to a patient-centric approach. Accordingly, given both the high unmet need across populations of patients with SMA and changing treatment paradigms, RESILIENT includes a broad population of participants to allow for generalization of the study findings. Participants were included irrespective of their SMA type or SMN upregulator background therapy. RESILIENT also includes both ambulant and nonambulant participants [5]. RESILIENT includes participants who currently are on a stable regimen of at least 1 SMN upregulator (i.e., have received the gene replacement therapy onasemnogene abeparvovec, which is administered as a single-dose intrathecal treatment and/or oral risdiplam administered daily and/or nusinersen administered intrathecally every 4 months). Individuals receiving treatment with a combination of SMN upregulators are eligible for participation. At the time of enrollment, participants in RESILIENT were between 4 years and 21 years of age. Table 1 summarizes the key eligibility criteria for RESILIENT.

#### 4.1.2. Interventions

During RESILIENT, taldefgrobep or matching placebo is administered subcutaneously once weekly. Participants weighing 15 kg to 40 kg receive 35 mg taldefgrobep or matching placebo and those weighing more than 40 kg receive 50 mg taldefgrobep or matching placebo. Body weight is monitored over the course of the study and weight-based dose adjustments are made accordingly. 

#### 4.1.3. Efficacy Outcomes and Safety Endpoints

The primary efficacy endpoint for RESILIENT is change in the 32-item Motor Function Measure (MFM-32) total score from baseline to week 48 of the study. The MFM-32 was selected as the primary endpoint because it performs well across a broad population, without the limitations demonstrated by alternative instruments. To date, the MFM-32 has been utilized as the primary endpoint in pivotal [63,64] and successful registrational trials in SMA [15]. The MFM-32 has also demonstrated benefit in both ambulant and nonambulant participants with SMA in a phase 2 dosing trial [21]. Covering a full spectrum of disease severity, MFM-32 was developed for evaluation of neuromuscular diseases, including SMA, in patients aged 6 years to 60 years [65]. The reliability and validity of the MFM-32 in patients with SMA type 2 and nonambulant SMA type 3 have been established, including test-test reliability, internal consistency reliability, convergent and known groups validity and responsiveness and magnitude of intra-individual clinical change [66,67,68]. Furthermore, the MFM-32 has demonstrated suitability for evaluating longitudinal change in both ambulant and nonambulant patients in natural history studies of SMA [7,69,70] and does not foster clustering of scores around ends of the scale, thus limiting the possibility of floor or ceiling effects when the full test is utilized [65,66,71].

Secondary efficacy endpoints in RESILIENT include the Revised Hammersmith Scale (RHS) and the Revised Upper Limb Module (RULM) [72,73,74,75,76,77,78] The RULM is specifically designed to assess upper limb function in individuals with SMA and has been shown to have good reliability, validity and item fit with little item overlap [79]. The RULM has been widely used in long-term longitudinal studies of up to 3 years in SMA [72,79,80] and was recently used as a secondary endpoint for identifying a difference between treated and untreated patients in a successful registrational trial in SMA [15,73]. In nonambulant populations, the RULM does not have a ceiling or floor effect [74,75,79]. 

The RHS is an SMA-specific scale that assesses physical abilities in individuals with SMA, ranging from weak SMA type 2 to strong ambulant SMA type 3 [76]. The RHS was designed to improve upon limitations of the Hammersmith Functional Motor Scale Expanded (HFMSE) and has indeed demonstrated reduced floor effect in an untreated cohort of patients with SMA type 2 or type 3 who were followed for 2 years [76,77]. The RHS offers good item fit, intra- and inter-rater reliability and construct and concurrent validity and clinically differentiates between groups, including groups based on SMA types, World Health Organization motor milestone categories and ambulatory status [76,78]. 

Other measures assessed in RESILIENT include (1) the self- and proxy-reported SMA Independence Scale–Upper Limb Module, which examines the level of assistance required to perform and participate in activities of daily living and correlates with the level of independence in daily life, including self-care [81] and (2) the Assessment of Caregiver Experience with Neuromuscular Disease, which assesses and quantifies the caregiver impact experienced by parents raising children with neuromuscular disease [82]. Additional assessments in RESILIENT include analyses of biomarkers and analyses of various Dual-Energy X-ray Absorptiometry (DXA) Scan derived measures such as total lean body mass. Safety measurements include assessment of AEs, vital signs, ECGs, physical measurements and examinations and clinical laboratory evaluations.

#### 4.1.4. Participant Timeline

RESILIENT is comprised of a screening period; a 48-week, double-blind phase in which participants are randomized to receive weekly, weight-based, blinded doses of taldefgrobep alfa (35 mg or 50 mg) or matching placebo; and an optional, 48-week, open-label extension available to eligible participants, during which all participants will receive taldefgrobep. After the baseline clinic visit, participants attend site visits at the clinic approximately every 12 weeks [83].

The first participant was enrolled in RESILIENT on 6 July 2022 and enrollment was completed on 14 September 2023. The double-blind phase of the study is estimated to conclude in the second half of 2024 [61,62].

#### 4.1.5. Study Procedures

The first phase of RESILIENT is being performed in a double-blind manner. Both the investigational product, taldefgrobep alfa solution for injection and the visibly indistinguishable placebo solution are dispensed in prefilled single-use safety syringes to allow for SC administration.

#### 4.1.6. Statistical Methods and Sample Size

The primary endpoint, change in the total MFM-32 score from baseline to week 48 of the double-blind period, will be analyzed with a mixed model for repeated measures. Statistical significance of differences in the primary endpoint between the taldefgrobep and placebo groups will be determined on the basis of a 2-sided alpha level of 0.05. The secondary endpoints of change from baseline to week 48 in RHS and RULM scores will be analyzed using the same methodology as that used for the primary endpoint. A total of 269 participants were enrolled in the study.

#### 4.1.7. Ethics

The RESLIENT protocol and any subsequent amendments were approved by the Independent Review Board or Independent Ethics Committees at each investigational site prior to initiation of the study. Furthermore, RESILIENT is being conducted in compliance with the study protocol, the recommendations prescribed in the Declaration of Helsinki and guidelines of the International Conference on Harmonization, Good Laboratory Practice and Good Clinical Practice.

## 5. Discussion

A comprehensive preclinical and clinical development plan for taldefgrobep was devised with the aim of demonstrating safety and efficacy in a broad population of those living with SMA who are treated with disease-modifying therapy. This manuscript highlights the preclinical and clinical development of taldefgrobep, explores the role of myostatin in muscle and introduces the phase 3 RESILIENT trial of taldefgrobep in SMA.

Prior to its consideration as a treatment for SMA, taldefgrobep was investigated in preclinical and clinical studies in both healthy adults and boys with DMD. Taldefgrobep demonstrated impressive tolerability in both the phase 1/2b and phase 2/3 investigations, consistent with its safety profile in healthy adults [41]. However, a futility analysis based on the North Star Ambulatory Assessment primary endpoint (https://clinicaltrials.gov/study/NCT03039686?term=NCT03039686&rank=1, accessed on: 1 September 2024) revealed that the total score at week 48 did not show statistically significant treatment differences in the intent-to-treat phase 2/3 population and the study was terminated early [41]. 

Several lessons can be applied from the experience with taldefgrobep in DMD. First, DMD is a disease in which absent or truncated dystrophin protein makes muscle fibers more susceptible to damage during contraction. As a result, fibrous tissue and fat replace muscle tissue over time, leading to less muscle tissue that is available for repair or amenable to improvement by targeted therapies [84,85]. In the phase 1b/2 and 2/3 trials of taldefgrobep in DMD, the mean age of participants at baseline was 8 years. It is possible that these participants had already experienced significant muscle degeneration at the time of treatment and earlier intervention could have demonstrated greater benefit [41]. Second, corticosteroids are widely used in DMD to slow or halt muscle weakness and delay loss of ambulation [84]. In this setting, corticosteroids are believed to abate symptoms through processes that include both reduction of inflammation and short-term promotion of muscle contractility [86]. However, these benefits are counterbalanced by the detrimental effects of long-term steroid use on muscle as well as inhibition of protein synthesis and catabolic protein breakdown, which may contribute to muscular atrophy [84]. Lastly, for most forms of DMD, there is a lack of disease-modifying therapies that can target the physiological processes underlying muscular degeneration. It is possible that taldefgrobep may offer enhanced outcomes if administered adjunctively with disease-modifying therapies, such as epylisin (FDA-approved in September 2016) and golodirsen (FDA-approved in December 2019), in patients for whom efficacy of these treatments has recently been established. However, neither of these disease-modifying drugs was FDA approved at the initiation of the phase 1b/2 studies of taldefgrobep in DMD and epylisin had been approved for only 9 months when patient recruitment for the phase 2/3 study in DMD began in 2017. Nevertheless, further preclinical studies in appropriate models would be required to test this hypothesis prior to initiating clinical trials [41,87,88]. 

Investigation in SMA is differentiated for a number of reasons. Specifically: SMA is a disease involving intact muscle, corticosteroids are not standard of care in SMA and SMN upregulators, currently approved by the FDA, help treat the root cause of SMA. Indeed, SMA is the first disease for which myostatin inhibitors are used after treatment of the primary genetic underlying condition. 

Newborn screening (NBS) programs for SMA are increasingly being implemented worldwide [89,90], allowing for rapid treatment of newly diagnosed patients. Nonetheless, there remain several critical unmet needs related to specific patient populations including but not limited to: (1) newborns with SMA who are not identified by NBS because they have a point mutation rather than a homozygous deletion that is detectable by polymerase chain reaction, (2) patients with two copies of *SMN2* and early symptomatic manifestations of SMA, (3) patients with SMA who were born before the implementation of NBS for this disease and (4) patients with SMA who continue to have functional impairments and reduced quality of life. Myostatin inhibitors are among several approaches being developed at the preclinical and clinical stage to address the needs of patients in these populations, who were potentially eligible for participation in RESILIENT [91]. 

In RESILIENT, the choice between using the MFM-32 or the HFMSE as the primary endpoint was carefully considered, as both scales have been used as primary endpoints in other successful therapeutic developments (NCT02292537, NCT02908685). However, the HFMSE has several limitations. Rasch analysis has identified high levels of differential item functioning with the HFMSE, indicating lack of measure stability across different patient groups and, consequently, challenges with validity in measuring motor performance in different SMA phenotypes [76,92]. Also, the HFMSE may be more susceptible to ceiling effects in stronger patients after successful therapeutic interventions [76]. In a successful registrational trial in SMA that utilized the MFM-32 as the primary endpoint, the HFMSE failed to identify a difference between treated and untreated patients, potentially as a result of floor effects [15]. Although the HFMSE may be appropriate for assessing stronger nonambulant individuals, it is potentially less sensitive in detecting changes among weaker populations [15]. Furthermore, as some items on the HFMSE are assessed with patients in the prone position, these items cannot be assessed in individuals who have undergone spinal fusion or have substantial hip flexor contractures [15].

The ultimate decision to use the MFM-32 as the primary endpoint in RESILIENT was based on the need to assess a broad population of patients with diverse levels of disease severity. The MFM-32 includes items to measure head, trunk, lower and upper limb and distal motor function, which is critically important in patients with severe SMA as these functions are preserved until the disease becomes advanced. Additionally, items in the MFM-32 and RULM that assess distal upper limb motor function can overcome floor effects of the HFMSE [15]. The MFM-32 can discriminate between ambulant vs nonambulant patients and between individuals with SMA type 2 vs type 3. The MFM-32 can also be effectively used as a measurement tool in patients as young as 2 years of age, regardless of SMA type or ambulant status. Even in very weak patients, the MFM-32 has the capacity to capture motor function changes, such as axial mobility and proximal or distal patterns of weakness. The MFM-32 also is capable of identifying functional declines occurring over 1 to 2 years. Additionally, the MFM-32 has been used jointly with other outcome measures in the development of other treatments, including risdiplam [64,93] and has consistently demonstrated good capacity for capturing functional changes over time.

In conjunction with the core elements of the RHS and RULM, as previously highlighted, the utilization of these combined measures as secondary endpoints in RESILIENT offers its own strengths. The RULM provides greater sensitivity for participants who score from 1 to 20 on the RHS. Thus, when used together with the RHS, the RULM enhances the ability of the RHS to assess disease progression in weaker patients [77]. The RHS was selected as a secondary rather than primary endpoint in RESILIENT because there are limited published data on the magnitude of clinically meaningful change in this measure. Furthermore, although the RHS is currently being used in a phase 2/3 clinical trial of the investigational compound RO7204239 (NCT05115110), to date there are no published data on how the RHS performs in such pivotal clinical trial settings, making it difficult to fully assess its strengths and limitations.

Additional exploratory analysis of biomarkers and outcome measures will be included in the final analysis. 

## 6. Future Directions

There remains a high unmet need in those living with SMA who continue to have functional impairments and reduced quality of life. Current therapies all upregulate SMN protein production, but none target the muscle. Taldefgrobep is a novel myostatin inhibitor that specifically targets signaling though the receptor, with competitive inhibition of activin A and myostatin. Combining myostatin inhibition with SMN upregulation offers promise in SMA. Given a robust scientific and clinical rationale and the favorable safety profile of taldefgrobep in patients with neuromuscular disease, the RESILIENT phase 3, randomized, placebo-controlled trial is investigating taldefgrobep as an adjunct to SMN upregulators in SMA (NCT05337553). 

The MFM-32, the primary endpoint in RESILIENT, is currently a sound measure for determining clinical efficacy in SMA. Despite the reliability and sensitivity of this validated measure, intrinsic qualities of participants or therapists, such as motivation, can introduce inter-rater variability in scoring the MFM. Future use of technologies that allow for markerless motion capture and analysis will allow for greater standardization of this key efficacy measure [94]. Wearable devices, such as those that have been explored in diseases like DMD [95,96,97] and eventually qualified as a primary endpoint in clinical trials, [98] offer promise in SMA, not only to enable individuals to monitor disease [99] but also to potentially support activities of daily living, such as gait [100].

Certain subpopulations of those living with SMA may particularly benefit from the combination of an SMN upregulator and taldefgrobep. The findings from RESILIENT will be informative to guide study into a broader population of SMA patients. NBS has proven to be a cost-effective measure, with lower financial burden for treated patients identified by early screening than for those who begin treatment after symptom onset [101,102]. Approximately 96% of patients can be diagnosed with NBS; those with heterozygous mutations are still not yet identified and present with a very significant clinical unmet need [102]. Likewise, newborns diagnosed with SMA via NBS and treated with SMN upregulators after birth experience improved outcomes compared to patients treated later in the course of disease after symptoms have manifested [102]. It is possible that introducing myostatin inhibition in combination with SMN upregulators in populations at an earlier stage of disease may amplify this treatment effect. Individuals with two *SMN2* copies may also be a population of focus in the future, as these patients have lower rates of survival compared to patients with three or four *SMN2* copies and thus have a greater unmet need [103,104].

The novel mechanism of blocking not only myostatin but other TGFB ligands, including Activin A, along with patient convenient SC administration avoiding travel for medically administered infusion, positions taldefgrobep alfa as an attractive potential therapy for patients that have limited mobility. 

In the future, it may be beneficial for researchers to study myostatin inhibitors such as taldefgrobep in other neuromuscular diseases. The use of gene therapy in congenital myopathies, including x-linked myotubular myopathy (NCT03199469), is being investigated in clinical trials. It is possible that, similar to the combination of myostatin inhibition with SMN upregulation in SMA, agents like taldefgrobep used in combination with gene therapy could further support skeletal muscle growth. Clinical trials would be required to validate this hypothesis.

## Figures and Tables

**Table 1:** RESILIENT key eligibility criteria. ECG, electrocardiogram; MAGEC, Magnetic Expansion Control; *SMN2*, survival motor neuron 2 [5].

Key Inclusion Criteria [5]	Key Exclusion Criteria [5]
4–21 years of age Body weight ≥ 15 kg Diagnosis of 5q autosomal recessive SMA with *SMN2* copy number confirmed by genetic testing Ambulant or nonambulant Stable on risdiplam and/or nusinersen for ≥6 months and/or history of ≥1 dose of onasemnogene abeparvovec received ≥2 years prior to screening and expected to remain on the same regimen throughout the study MFM-32 total score < 90% out of 100% (achieved by a total mark raw score of ≤86 out of 96) at screening Life expectancy of >2 years at screening (based on investigator’s judgment)	Prior anti myostatin therapies History of spinal fusion or major surgeries within 6 months prior to screening or planned during the study (nonsurgical adjustments, such as MAGEC rods, allowed) Implanted shunt for cerebral spinal fluid drainage or implanted central nervous system catheter Need for invasive or noninvasive ventilation for daytime treatment to maintain respiratory sufficiency (use during daytime naps or overnight allowed) History/evidence of organ dysfunction or significant deviation from normal range on vital signs, physical examination, ECG, or clinical laboratory values that exceeds what is consistent with the target population Hypersensitivity to taldefgrobep alfa (ascertained or presumed) or history of severe allergy to biological therapies

## Data Availability

Data will be made available upon reasonable request. The data sets used and/or analyzed during the current study will be available from the corresponding author upon reasonable request.
